# Prioritizing Land and Sea Conservation Investments to Protect Coral Reefs

**DOI:** 10.1371/journal.pone.0012431

**Published:** 2010-08-30

**Authors:** Carissa J. Klein, Natalie C. Ban, Benjamin S. Halpern, Maria Beger, Edward T. Game, Hedley S. Grantham, Alison Green, Travis J. Klein, Stuart Kininmonth, Eric Treml, Kerrie Wilson, Hugh P. Possingham

**Affiliations:** 1 The Ecology Centre, University of Queensland, St. Lucia, Queensland, Australia; 2 Australian Research Council Centre of Excellence for Coral Reef Studies, James Cook University, Townsville, Queensland, Australia; 3 National Center for Ecological Analysis and Synthesis, Santa Barbara, California, United States of America; 4 The Nature Conservancy, Brisbane, Queensland, Australia; 5 Queensland University of Technology, Brisbane, Queensland, Australia; 6 Australian Institute of Marine Science, Townsville, Queensland, Australia; NIWA, New Zealand

## Abstract

**Background:**

Coral reefs have exceptional biodiversity, support the livelihoods of millions of people, and are threatened by multiple human activities on land (*e.g.* farming) and in the sea (*e.g.* overfishing). Most conservation efforts occur at local scales and, when effective, can increase the resilience of coral reefs to global threats such as climate change (*e.g.* warming water and ocean acidification). Limited resources for conservation require that we efficiently prioritize where and how to best sustain coral reef ecosystems.

**Methodology/Principal Findings:**

Here we develop the first prioritization approach that can guide regional-scale conservation investments in land- and sea-based conservation actions that cost-effectively mitigate threats to coral reefs, and apply it to the Coral Triangle, an area of significant global attention and funding. Using information on threats to marine ecosystems, effectiveness of management actions at abating threats, and the management and opportunity costs of actions, we calculate the rate of return on investment in two conservation actions in sixteen ecoregions. We discover that marine conservation almost always trumps terrestrial conservation within any ecoregion, but terrestrial conservation in one ecoregion can be a better investment than marine conservation in another. We show how these results could be used to allocate a limited budget for conservation and compare them to priorities based on individual criteria.

**Conclusions/Significance:**

Previous prioritization approaches do not consider both land and sea-based threats or the socioeconomic costs of conserving coral reefs. A simple and transparent approach like ours is essential to support effective coral reef conservation decisions in a large and diverse region like the Coral Triangle, but can be applied at any scale and to other marine ecosystems.

## Introduction

Coral reefs are the world's most diverse marine ecosystem and are vital to hundreds of millions of people as a source of nutrition, economic opportunity, and storm protection [1]. Due to climate change and local impacts, the state of coral reefs is grim and their protection is urgent [2], [3], [4]. As with all conservation, limited resources for coral reef protection require that we prioritize where and how to act to efficiently sustain coral reef ecosystems [5].

Local-scale threats to coral reefs originate from both land- and sea-based human activities (*e.g.* over-fishing, nutrient runoff from farming) [6]. Where both exist, conservation strategies should consider each of them [7], [8]. The allocation of conservation resources to coral reefs should depend on which strategies most efficiently reduce their threats [9]. Sophisticated approaches for identifying marine conservation priorities exist [7], [10], [11], but fail to explicitly address threats originating on land and the associated costs of mitigating these threats through conservation action. Effective conservation prioritization should provide guidance on how to distribute funds between land- and sea-based conservation actions to protect coral reefs.

We address this deficiency by developing the first explicit method for prioritizing conservation actions and locations to cost-effectively mitigate land- and sea-based threats to marine ecosystems and apply it to the Coral Triangle, one of the world's highest conservation priorities [6], [12]. The multi-lateral Coral Triangle Initiative on Coral Reefs, Fisheries and Food Security (formalized in May 2009) is the focus of significant global conservation attention with financial commitments of at least US $400 million (http://www.cti-secretariat.net). This amount is likely to be insufficient to achieve the Initiative's goals, thus investments must be prioritized.

Standard advice from business and economics is to invest in projects where the rates of return on investment are the highest [13]. This approach has been applied to the conservation of terrestrial biodiversity [13], [14], [15], but it has yet to be applied to marine conservation. Any application of the return on investment approach requires an explicit statement of overall objective. The objective in previous studies has been species focused (*e.g.* maximize the number of species conserved). Here, our objective is to maximize threat reduction to coral reefs across the Coral Triangle's ecoregions through investment in land- and sea-based conservation actions.

Achieving the objective relies upon a rigorous problem formulation and information on threats to marine ecosystems, effectiveness of management actions at abating threats, and the economic costs of actions. We considered 8 threats to coral reefs (Table 1), each associated either with agricultural run-off or fishing, and two actions that reduce their impact on coral reefs: effective management of coastal watersheds and coral reefs. We refer to the places where these actions are implemented as protected areas, but acknowledge that effective management is rare and involves more than just dedicating protected areas, especially in the Coral Triangle [16], [17], [18]. As a result, protected areas require funding for management and the restriction of profitable activities. Thus, we estimated the management costs and value of foregone usage to farmers and fishers (*i.e.* opportunity cost) of protected areas.

**Table 1:** Threats and their relative impact on coral reef ecosystems.

Threat	Relative impact ( 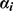 )
Nutrient run-off from fertilizers	1.8
Organic pollution run-off from pesticides	1.2
Artisanal fishing	2.3
*Commercial fishing*	
Demersal, destructive	1.2
Demersal, non-destructive, high bycatch	1.6
Demersal, non-destructive, low bycatch	1.3
Pelagic, high by-catch	0.5
Pelagic, low by-catch	0.7

The relative impact values were determined from an expert-based survey [6], [20].

Using this information, we calculated the rate of return on investment of each action in each ecoregion (denoted “ecoactions”) [13], where the rate is calculated as the reduction of threats (return) per dollar spent on their reduction (investment). We ranked each ecoaction (*e.g.* effective management of coral reefs in the Bird's Head ecoregion) in terms of how cost-effective it is at mitigating threats to coral reefs for two scenarios. Scenario 1 reflects investment of management costs alone, whereas scenario 2 also considers opportunity costs. We demonstrate how these rankings can be used to allocate a limited budget for conservation and we compare our results to those based on individual criteria (*e.g.* cost, species richness).

## Materials and Methods

**Figure 1:**
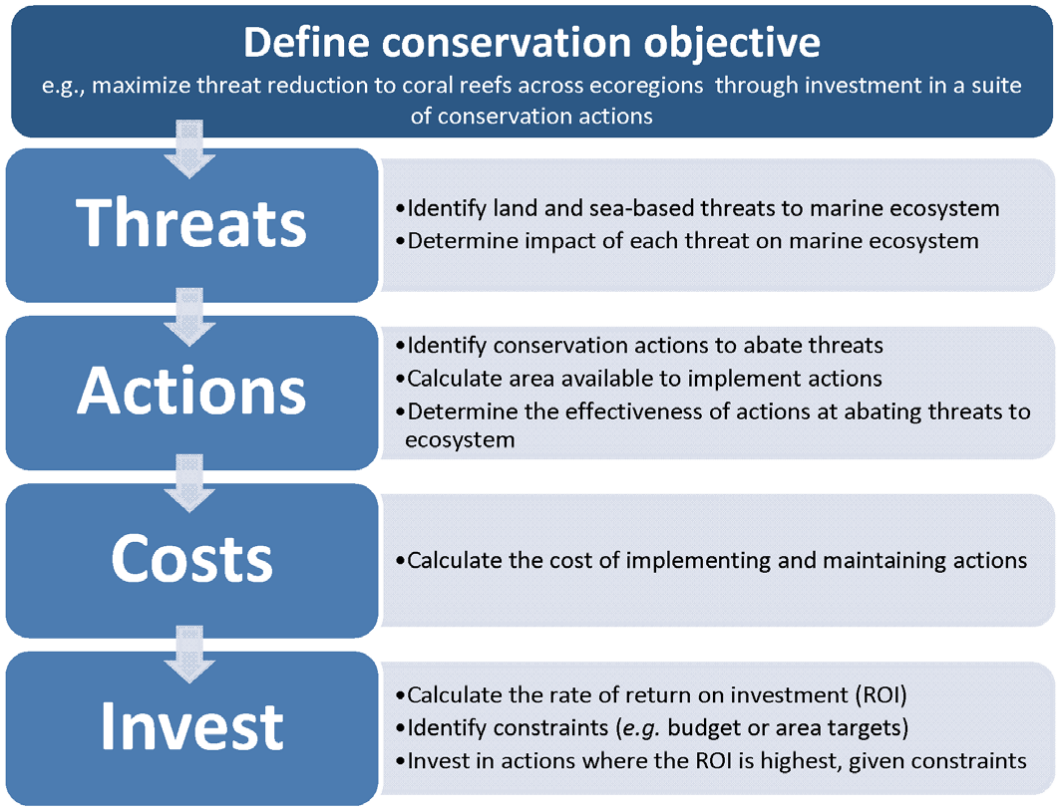
Resource allocation method for prioritizing among land and sea-based conservation actions and locations.

Our method for prioritizing land and sea conservation investments to protect marine ecosystems involves five general steps (Figure 1), described below.

### Step 1: Define conservation objective

The first step in formulating any conservation resource allocation problem is to define a quantifiable objective. Our objective was to maximize threat reduction to coral reefs across the Coral Triangle's ecoregions through investment in land and sea-based conservation actions. We used 16 marine ecoregions that were defined on the basis of coral diversity and endemism, each of which contains 503–553 zooxanthellate coral species [19].

### Step 2: Identify threats to ecosystem

The second step is to determine the threats to, and their relative impact on, the marine ecosystem. We considered the threats that could be mitigated with local-scale conservation action and their relative impact on coral reefs (Table 1). We used data from Halpern *et al.* [6] that depicts the impact of anthropogenic drivers of change (henceforth referred to as threats), to each 1 km^2^ section of coral reefs [6], [20].

### Step 3: Identify conservation actions to abate threats

The third step is to identify conservation actions and their effectiveness at abating the threats identified in step two. We determined the area available (*i.e.* not cleared or effectively managed in each ecoregion) for implementing two actions (Table S1): 1) effective management of coastal watersheds; 2) effective management of coral reefs [18]. We assume that each threat reduces linearly with protection of the ecoregion.

A surrogate must be used to represent where and how much of the land and sea is effectively managed at present, as this information does not exist across the Coral Triangle. In theory, protected areas are effectively managed; however, in practice, only a subset of protected areas is effectively managed for biodiversity conservation [16], [18]. Therefore, we estimated which coral reefs and terrestrial protected areas are effectively managed based on a few simple guidelines, described below.

#### Land-based conservation

We only considered sub-catchments that reach the ocean and consider them as part of an ecoregion if their coastal pour-point emptied into the marine portion of that region. We used sub-catchment boundaries and coastal pour-point data from Halpern *et al.* [6]. We assume that the protected areas are effectively managed in areas containing native vegetation. In this analysis, we used terrestrial protected areas with an IUCN designation from the World Database on Protected Areas from the World Commission on Protected Areas from December 2007. Using SPOT vegetation satellite data, we determined the amount of protected areas containing native vegetation from 2000 [21]. For each ecoregion, we calculated the proportion of land protected under two scenarios: 1) Pessimistic scenario, where vegetated areas in only the more stringently protected areas (*i.e.* IUCN 1-4) are effective and 2) Optimistic scenario, where vegetated areas in all types of protected areas recognized by the IUCN (IUCN 1-6) are effective.

#### Marine conservation

We used the global coral reef atlas [22], compiled by the World Conservation Monitoring Centre at the United Nations Environment Programme, to determine the location of coral reefs with each ecoregion. Data indicate the presence/absence of coral reefs for each 1 km^2^ cell. Mora *et al.* [18] provided an assessment on the extent and effectiveness of coral reef protected areas. Each protected area was classified by its regulations on extraction (no-take, take, or multi-purpose) and risk of poaching (low, medium, high). For each ecoregion, we calculated the proportion of coral reef protected under two scenarios: 1) Pessimistic scenario, where only areas with no extraction (no-take, low poaching) are effective at protecting the reefs and 2) Optimistic scenario, where areas with limited extraction (no-take or multipurpose for any level of poaching) are effective at protecting the reefs.

We show results that use the amount protected under the pessimistic scenario for terrestrial and marine conservation. However, the ranking results were insensitive to the information used as there is little difference between the amounts protected under each scenario.

### Step 4: Calculate costs of implementing actions

We predicted the annual management and opportunity costs associated with land and marine protected areas (Table S1). When applying the method with opportunity costs, we assume that the ecoaction excludes extractive activities and causes economic losses that cannot be recovered in another place or industry. However, in reality, conservation can deliver benefits (*e.g.* improved fishing yields) that may compensate for some economic losses [23].

#### Management costs

We used a model developed by Moore *et al.* [24] to predict the management costs of terrestrial protected areas in each ecoregion, as done in Kark *et al* [25] and Bode *et al* [26]. The model states that the cost of managing a protected area is a nonlinear function of the size of the proposed protected area, the Purchasing Power Parity (PPP) of the nation, and the Gross National Income (GNI) of the nation:
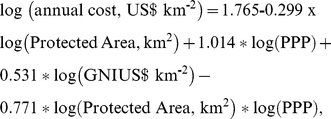



where all logarithms are of base ten. We modeled the area of protected areas in each ecoregion by the median size of the existing vegetated protected areas (IUCN 1-6) in that ecoregion.

We used a model developed by Balmford *et al.* [27] to predict the management costs of marine protected areas in each ecoregion. The model states that the cost of managing a marine protected area is a nonlinear function of the size of the proposed protected area, distance of area from land, and the PPP of the nation:
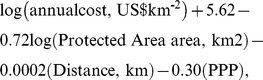



where all logarithms are of base ten. We modeled the area of protected areas in each ecoregion by the median size of the existing no-take or multipurpose coral reef protected areas in that ecoregion. We modeled the distance of coral reef protected areas in each ecoregion by the median distance of coral reefs from land.

The economic data we used to inform the models described below were obtained from the 2006 International Monetary Fund's Financial Statistics http://www.imfstatistics.org/imf/. To calculate the PPP, we divided the PPP conversion rate (local $/international $) reported by the Monetary Fund by the exchange rate (local $/US $). We substituted missing GNI information with the Gross Domestic Product.

As some ecoregions span multiple countries, the management costs are therefore likely to vary substantially. Our analyses treat each region as a homogeneous entity, where the cost is calculated using the Balmford-Moore models [24], [27], with parameter values that are the area-weighted average of the constituent nations' exclusive economic zone. The area-weighting method is applied to the other predictor variables as done in Bode *et al* [26].

#### Opportunity costs, land

We estimated the opportunity costs of agricultural production from implementation of a protected area that excludes cultivation. The agriculture opportunity cost represents the potential foregone economic returns from agricultural production (cropping and grazing) on areas containing native vegetation [28]. The potential economic returns from agricultural production are estimated at a 5′ resolution by the maximum of the potential crop and livestock yields based on land capability, multiplied by the producer price [28]. For each ecoregion, we calculated the maximum potential agricultural profits per unit area of native vegetation.

#### Opportunity costs, marine

We estimated the lost opportunity costs to fishermen (*C*) from implementation of a coral reef protected area that excludes fishing:
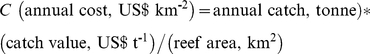



Spatially explicit information on catch rates for small-scale fisheries was determined for each 1 km^2^ of coral reef by Halpern *et al.* [29] from the FAO and Sea Around Us Project (SAUP). Although this is the best available data for artisanal fishing, it is modeled and based on many crude assumptions. Development of a new artisanal fishing model for the Coral Triangle that considers the spatial distribution of catch, population size of species across the region, historical fishing, and fishing method is an area of further research. We summed the catch rates across all coral reefs within each ecoregion. The value (US$, year 2000) of reef fish in each country are provided by the SAUP for reported landings from 1950–2004 [30]. We used the maximum value reported per country to prevent underestimating the value over time. Like management costs, opportunity costs in some ecoregions vary substantially because they span multiple countries. Our analyses treat each region as one entity using the area-weighted average of the constituent nation's exclusive economic zone.

### Step 5: Invest where the rate of return on investment is highest

The final step is to mathematically formulate the resource allocation problem and determine the rate of return (*i.e.* reduction of threats) on investment (*i.e.* cost of reducing threats) of each ecoaction. The overall impact, *I_i_*, that a set of threats (*k* = 1,…,8) have on a 1 km^2^ section of coral reef (*i = *1,…,N*)* was defined by Halpern *et al.* [29] as a weighted sum of land- and sea-based threats
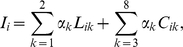
where *L_ik_* and C*_ik_* are threat values originating from the land and sea, respectively, and 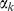 is a weighting reflecting the relative impact of threat *k* on coral reefs (Table 1).

In step 3, we made the assumption that threat, *k*, in any 1 km^2^ section of reef, *i*, is reduced linearly with protection of the ecoregion, *j*: 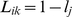 and 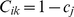, where *l_j_* and c*_j_* are the proportion of terrestrial and coral reef protected area, respectively. Therefore, the average threat impacting coral reefs in each ecoregion (*j* = 1,…,16) can be written as a function of how much of the land and sea that we protect in an ecoregion,
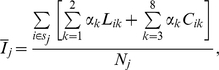
where *N_j_* is the number of reef pixels (*i*) in ecoregion *j* and *S_j_* is the set of indices that determine if pixel *i* is in region *j*. In doing this, we assume that the benefit of protection is evenly spread across the ecoregion. This relationship could be modified if more discrete regions were targeted.

The proportion of the ecoregion protected is the sum of the portion currently protected (*l_oj_* and *c_oj_*) and the portion protected by additional investment. To account for the cost of additional protection, the proportion protected after additional investment made can be expressed as the proportion of additional investment made (*x_j_* and *y_j_*) relative to the total cost of land and ocean available for protection (*a_j_* and *b_j_*), respectively:


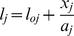
 and 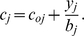


The rate of return (threat reduction) on investment of each ecoaction can then be calculated for each land and sea-based conservation action, respectively:


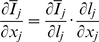
 and 
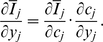


The greater the rate of return on investment per ecoaction, the higher priority it is for investment. In order to achieve the conservation objective, investments should be made in high priority ecoactions unless there are ecoregional or action-specific constraints (*e.g.* budget or area targets). We show how a budget and area constraint influences the distribution of an arbitrary budget of US $1 B, $400 M, and $100 M. The area constraint ensures that a priority ecoaction receives funding for no more than a designated percentage of its available area, which we arbitrarily selected to be 30%.

## Results

### Ranking

**Figure 2:**
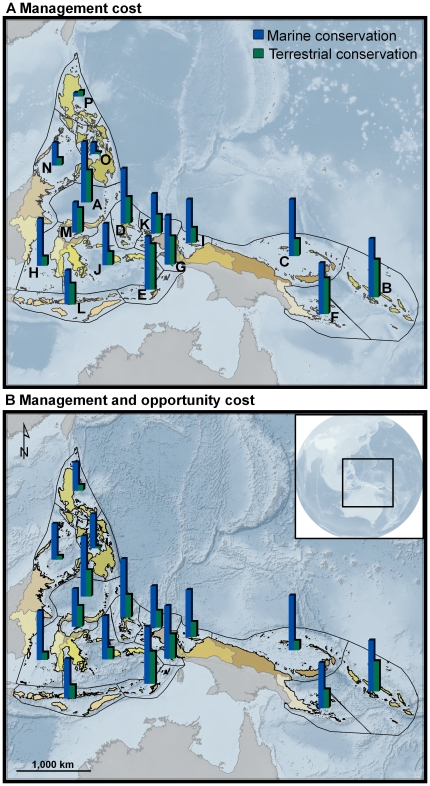
Ecoaction rankings indicate their relative priority for coral reef conservation investment across the Coral Triangle (taller bar, higher rank). Scenario 1 (a) reflects investment of management costs whereas scenario 2 (b) also considers opportunity costs. Letters labeling ecoregions follow the ranking order for marine conservation (*i.e.* Ecoregion A ranks highest for marine conservation) and correspond to letters in Figure 3.

We applied our prioritization approach to rank ecoactions using different costs and found a high concordance in the rankings (Spearman's rank correlation of 0.88, p<0.001). We present our ranking results for both scenarios at two scales (Fig. 2): across the entire Coral Triangle and within each ecoregion. At the Coral Triangle scale, we found that terrestrial conservation in one ecoregion is sometimes a higher priority than marine conservation in another ecoregion, especially in scenario 1 (management costs only). For example, the highest ranking terrestrial action (E, North Arafura ecoregion) has a larger return on investment than marine conservation in half of the ecoregions.

**Figure 3:**
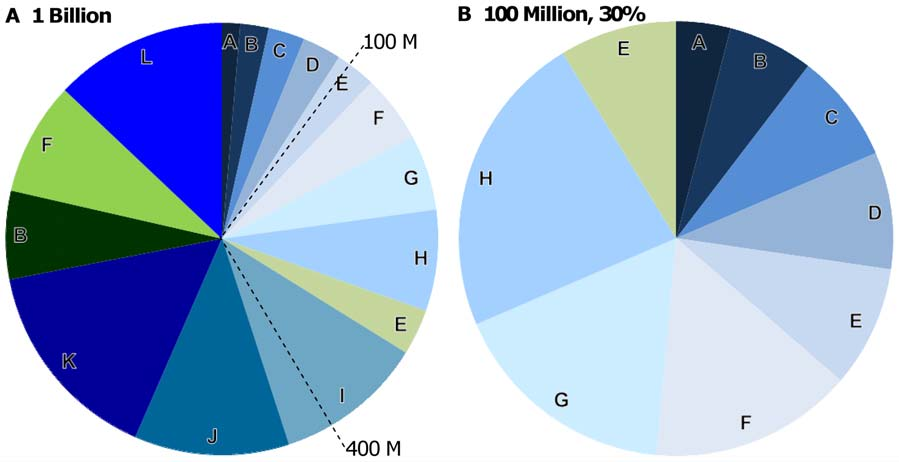
Distribution of an annual budget (*e.g.*, US $1 B, $400 M, and $100 M) to the highest ranking ecoactions. Funding is distributed to all available reef and land habitat (a) and restricted to 30% of available habitat (b) under scenario 1 (management costs). Shades of green and blue represent funding to land- and sea-based conservation, respectively. Letters correspond to ecoregion labels in Figure 2a.

Within any particular ecoregion, marine conservation is almost always a higher priority than terrestrial conservation. The one exception is in the North Philippines (Scenario 1), where the marine management cost is substantially larger than on the land (Table S1).

### Budget Allocation

We demonstrate two ways these rankings can be used to allocate limited conservation resources under scenario 1 (management costs) (Fig. 3). First, for three different budgets (US $ 1 B, 400 M, and 100 M), we allocate money to the highest ranking ecoactions until it is spent (Fig. 3a). This assumes that within an ecoregion, all available (*i.e.* not currently protected or developed) coral reefs and land can be effectively managed, which is likely to be unrealistic. Thus, we show how a budget would be distributed to the highest ranking ecoactions if we cap the allocation at protection of thirty percent of the available reef or land affecting the reef (Fig. 3b).

To explore the sensitivity of our results to the threat weighting values (Table 1), we performed the analysis with the range of weighting values provided by experts (n = 24) and found that the rankings were robust to these variations (Spearman's rank correlations >0.99, p<0.001). Regardless of weighting values used, rankings for the top seven ecoactions were always the same. The remainder of ecoactions typically did not change rank and never changed by more than four places (Table S2). Depending on the budget, how these subtle discrepancies could impact the distribution of funding are important considerations.

### Comparison

We compare our ecoregional rankings to those based on individual criteria (Table 2). Since other approaches do not consider marine and terrestrial conservation actions simultaneously, we compare our ecoregional rankings for marine actions only. We found a lack of concordance between approaches (Spearman's rank correlations from -0.22 to 0.3), indicating that they would recommend different investment priorities. Using estimated management costs, we show how a budget of US $400 M for management of land- and sea-based threats would be distributed following each ranking scheme (Table 2). For example, we found that prioritization on cumulative threats alone would only provide enough funding for effective management of 6% of one ecoregion, whereas our approach would ensure that 30% of seven ecoregions were managed.

**Table 2:** Comparison of marine priorities determined using different approaches.

Ecoregion	ROI	ROI	No. of coral reef		Annual costs		Avg. impact	
	Scenario 1	Scenario 2	species		(US $/km^2^)		(I/km^2^-reef)	
			Actual	Rank	Actual	Rank	Actual	Rank
A, Celebes Sea	1*	1*	545	2*	62,598	12	18.0	3
B, Solomon Islands	2*	6*	476	15	56,032	11	13.5	8
C, Bismarck Sea	3*	4*	500	14	34,059	1*	12.5	10
D, Halmahera	4*	2*	544	3*	48,836	8	15.0	7
E, North Arafura	5*	3*	519	9	63,149	13	12.0	11
F, Milne Bay	6*	9	475	16	43,898	3*	7.4	16
G, SW. Papua	7*	5*	540	4*	46,736	6	12.6	9
H, Makassar	8	7	511	12	49,646	9	10.5	14
I, Cenderawasih	9	8	515	11	46,562	5	15.3	5
J, Banda & Molluccas	10	10	533	6	51,828	10	10.5	13
K, Bird's Head	11	11	553	1*	46,241	4	11.8	12
L, N. Lesser Sunda & Savu	12	12	523	8	43,627	2*	17.1	4
M, Gulf of Tomini	13	13	518	10	48,130	7	10.2	15
N, Sulu Sea	14	14	540	4	114,645	14	15.1	6
O, SE. Philippines	15	15	533	6	427,893	16	26.0	2
P, N. Philippines	16	16	510	13	415,967	15	28.3	1

We compare rankings on the basis of 1) Return on investment (ROI) analysis for marine conservation for both scenarios; 2) Coral reef species richness; 3) Annual opportunity and management cost (lower cost, higher rank); 4) Average cumulative impact on coral reefs from all human activities [6]. Higher return, richness, and impact values were given a higher rank and equivalent values were assigned the same rank. The spatial location of the ecoregions is indicated by letter in Fig. 2.

*Thirty percent of the available reef or land affecting the reef would be effectively managed with a fixed budget of US $400 M.

## Discussion

The purpose of this paper is to illustrate a novel approach for delivering cost-effective outcomes for marine conservation that can explicitly trade-off resource allocation decisions among land- and sea-based conservation actions to protect marine ecosystems. Our approach is useful in guiding managers and policy makers in making decisions on where and in which actions to invest. Although it is useful for supporting broad scale resource allocation decisions, the results are not necessarily applicable to all places within an ecoregion as the conservation context may vary between communities [31]. However, the method can also be applied at a local-scale (*e.g.* provincial or catchment level), using more conservation actions (*e.g.*, run-off management, improved agricultural practices, fishing gear-based management). In addition, more specific data on social and economic costs would need to be estimated for a local-scale application as the data we used may be too coarse, especially for management costs. The effectiveness of any local conservation plan in this region is reliant upon community involvement and the consideration of indigenous knowledge, management practices, and property rights [31], [32], [33].

One of the key results - terrestrial conservation in one ecoregion is sometimes a higher priority than marine conservation in another ecoregion - is contrary to current conservation strategies, which typically do not trade-off marine and terrestrial conservation actions to protect marine ecosystems, and suggests that more cost-effective conservation outcomes could be achieved using our method. Although another key result – within any particular ecoregion, marine conservation is almost always a higher priority than terrestrial conservation within an ecoregion - generally supports current management practice in any given place, greater conservation outcomes could be achieved when the entire region is considered.

Incorporating different socioeconomic costs did not significantly affect outcomes. However, decisions following each scenario are likely to have different social and economic implications. For example, investments including opportunity costs (Scenario 2) are more likely to minimize impact on fishers and farmers as they were explicitly considered in the analysis [34], [35], [36]. However, scenario 2 assumes that people would be compensated for displacement due to conservation and that conservation actions preclude subsistence farming and fishing, both of which are unlikely.

We assume that each threat reduces linearly with protection of the ecoregion (Step 3, materials and methods). This represents the most parsimonious relationship between threat and protection but could easily be modified if more detailed information were available for each ecoaction. This type of information is difficult to obtain as effective monitoring and good quality data relevant to this is lacking [14]. However, in a region with little protection and a limited budget for conservation, the use of a non-linear function that demonstrates diminishing returns may not substantially impact the results. Testing this on a specific region where this type of information could be obtained would be informative. Assessing the benefits of conservation actions, including the relationship between reducing threats and biodiversity, is a significant challenge and research priority in conservation.

Other applications of the return on investment framework to inform the allocation of resources to protect terrestrial biodiversity use a non-linear benefit function based on the species-area relationship where the total number of species (*S*) present in area (*A*) is a power–law function of that area [13], [14]: 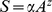. This relationship is an appropriate estimation of the benefits of protection when the objective is to conserve species, as in these studies; however, it is not applicable to our objective (*i.e.* threat reduction to coral reefs). Although we aimed to solve one objective, application of the return on investment thinking can be used to solve a range of conservation objectives to conserve marine ecosystems [13].

Priorities and investment plans following our approach versus that based on individual criteria (Table 2) would be substantially different. In addition, prioritization on species information alone, for example, will not be able to inform how funding should be divided between management actions on the land and in the sea. Similar confusion can arise if we prioritize only on cost or threat.

Our method could be adapted to provide more specific guidelines on how much and when (*i.e.* timing of investments) to invest in ecoactions [13]. Such analyses may require information on budget (size and constraints), benefits of conservation (*e.g.* payments for ecosystem services), more specific conservation actions, social adaptive capacity indicating the likelihood of a project succeeding (*e.g.* willingness of people to forego resources) [31], distribution of species, more opportunity costs (*e.g.* aquaculture and forestry), a better understanding of the effectiveness of management actions, coral reef resilience [37], and other relevant threats (*e.g.* sedimentation from deforestation). At any scale, neglecting to properly address social costs to resource users will most likely lead to unsuccessful conservation plans [38], [39]. These are areas of further research.

Although we apply our prioritization approach to the Coral Triangle Initiative, we acknowledge that our analysis is focused on a small aspect of the conservation problem in the Coral Triangle. In addition to identifying priority areas for effective management (Goal 1 in the Regional Plan of Action), the Coral Triangle Initiative aims to achieve outcomes relevant to fisheries management, climate change adaptation, and threatened species [40]. However, it is important to note that effective management of coral reefs at a local scale can increase their resilience to global threats such as climate change [9].

A simple, transparent, and economically grounded approach like ours is essential to making any conservation decisions in a large and diverse region like the Coral Triangle, where the budget is primarily financed from international aid. Effective conservation of marine resources must consider land- and sea-based human activities and their management costs [41]. The lack of a defensible resource allocation plan could lead to costly and contentious conservation strategies that do not protect biodiversity, impeding additional global funding to one of the world's most biodiverse and threatened regions.

## Supporting Information

Table S1 Cost and protected area data for coastal catchments and coral reefs in each ecoregion.
(0.04 MB DOC)

Table S2 Ranking results from scenario 1 compared to results from the impact weighting value sensitivity analysis. We perform the analysis with the maximum and minimum impact weighting values provided by experts for the land- and sea-based threats.
(0.05 MB DOC)

## References

[1] Dodge RE, Birkeland C, Hatziolos M, Kleypas J, Palumbi SR (2008). A Call to Action for Coral Reefs.. Science.

[2] Bellwood DR, Hughes TP, Folke C, Nystrom M (2004). Confronting the coral reef crisis.. Nature.

[3] Carpenter KE, Abrar M, Aeby G, Aronson RB, Banks S (2008). One-Third of Reef-Building Corals Face Elevated Extinction Risk from Climate Change and Local Impacts.. Science.

[4] Hughes TP, Baird AH, Bellwood DR, Card M, Connolly SR (2003). Climate change, human impacts, and the resilience of coral reefs.. Science.

[5] Bottrill MC, Joseph LN, Carwardine J, Bode M, Cook C (2008). Is conservation triage just smart decision making?. Trends in Ecology & Evolution.

[6] Halpern BS, Walbridge S, Selkoe KA, Kappel CV, Micheli F (2008). A global map of human impact on marine ecosystems.. Science.

[7] Halpern BS, Ebert CM, Kappel CV, Madin EMP, Micheli F (2009). Global priority areas for incorporating land & sea connections in marine conservation.. Conservation Letters.

[8] Richmond RH, Rongo T, Golbuu Y, Victor S, Idechong N (2007). Watersheds and coral reefs: Conservation science, policy, and implementation.. Bioscience.

[9] Baskett ML, Nisbet RM, Kappel CV, Mumby PJ, Gaines SD (2010). Conservation management approaches to protecting the capacity for corals to respond to climate change: a theoretical comparison.. Global Change Biology.

[10] Hughes TP, Bellwood DR, Connolly SR (2002). Biodiversity hotspots, centres of endemicity, and the conservation of coral reefs.. Ecology Letters.

[11] Roberts CM, McClean CJ, Veron JEN, Hawkins JP, Allen GR (2002). Marine biodiversity hotspots and conservation priorities for tropical reefs.. Science.

[12] Brooks TM, Mittermeier RA, Fonseca GABd, Gerlach J, Hoffmann M (2006). Global Biodiversity Conservation Priorities.. Science.

[13] Wilson KA, Underwood E, Morrison S, Klausmeyer K, Murdoch W (2007). Conserving biodiversity efficiently: What do do, Where and When.. PLoS Biology.

[14] Murdoch W, Polasky S, Wilson KA, Possingham HP, Kareiva P (2007). Maximizing return on investment in conservation.. Biological Conservation.

[15] Wilson KA, McBride M, Bode M, Possingham HP (2006). Prioritising global conservation efforts.. Nature.

[16] Bruner AG, Gullison RE, Rice RE, da Fonseca GAB (2001). Effectiveness of parks in protecting tropical biodiversity.. Science.

[17] Curran LM, Trigg SN, McDonald AK, Astiani D, Hardiono YM (2004). Lowland forest loss in protected areas in Indonesian Borneo.. Science.

[18] Mora C, Andrefouet S, Costello MJ, Kranenburg C, Rollo A (2006). Coral reefs and the global network of marine protected areas.. Science.

[19] Veron J, deVantier L, Turak E, Green A, Kinnimonth S (2009, in review). Global coral biodiversity: a blueprint for reef conservation.. don't know.

[20] Halpern BS, Selkoe KA, Micheli F, Kappel CV (2007). Evaluating and ranking the vulnerability of global marine ecosystems to anthropogenic threats.. Conservation Biology.

[21] Stibig HJ, Achard F, Fritz S (2004). A new forest cover map of continental southeast Asia derived from SPOT-VEGETATION satellite imagery.. Applied Vegetation Science.

[22] Spalding M, Ravilious C, Green E (2001). World Atlas of Coral Reefs..

[23] Russ GR, Alcala AC, Maypa AP, Calumpong HP, White AT (2004). MARINE RESERVE BENEFITS LOCAL FISHERIES.. Ecological Applications.

[24] Moore J, Balmford A, Allnutt T, Burgess N (2004). Integrating costs into conservation planning across Africa.. Biological Conservation.

[25] Kark S, Levin N, Grantham H, Possingham HP (2009). Between-country collaboration and consideration of costs increase conservation planning efficiency in the Mediterranean Basin.. Proceedings of the National Academy of Sciences.

[26] Bode M, Wilson KA, Brooks TM, Turner WR, Mittermeier RA (2008). Cost-effective global conservation spending is robust to taxonomic group.. Proceedings of the National Academy of Sciences.

[27] Balmford A, Gravestock P, Hockley N, McClean CJ, Roberts CM (2004). The worldwide costs of marine protected areas.. Proceedings of the National Academy of Sciences of the United States of America.

[28] Naidoo R, Adamowicz WL (2005). Economic benefits of biodiversity exceed costs of conservation at an African rainforest reserve.. Proceedings of the National Academy of Sciences of the United States of America.

[29] Halpern BS, Kappel CV, Selkoe KA, Micheli F, Ebert C (2009). Mapping cumulative human impacts to California Current marine ecosystems.. Conservation Letters.

[30] Sumaila UR, Marsden AD, Watson R, Pauly D (2007). A global ex-vessel fish price database: construction and applications.. Journal of Bioeconomics.

[31] McClanahan TR, Cinner JE, Maina J, Graham NAJ, Daw TM (2008). Cosnervation action in a changing climate.. Conservation Letters.

[32] Smith RJ, Verissimo D, Leader-Williams N, Cowling RM, Knight AT (2009). Let the locals lead.. Nature.

[33] Klein C, Steinback C, Scholz A, Possingham H (2008). Effectiveness of marine reserve networks in representing biodiversity and minimizing impact to fishermen: a comparison of two approaches used in California.. Conservation Letters.

[34] Klein C, Chan A, Kircher L, Cundiff A, Hrovat Y (2008). Striking a balance between biodiversity conservation and socioeconomic viability in the design of marine protected areas.. Conservation Biology.

[35] Carwardine J, Wilson K, Watts M, Etter A, Klein C (2008). Avoiding costly conservation mistakes: the importance of defining actions and costs in spatial priority setting.. Plos One.

[36] Ban N, Klein CJ (2009). Spatial socio-economic data as a cost in systematic marine conservation planning.. Conservation Letters.

[37] Gunderson LH (2000). Ecological resilience - in theory and application.. Annual Review of Ecological Systems.

[38] Sala E, Aburto-Oropeza O, Paredes G, Parra I, Barrera JC (2002). A General Model for Designing Networks of Marine Reserves.. Science.

[39] Klein C, Steinback C, Watts M, Scholz A, Possingham H (2010). Spatial marine zoning for fisheries and conservation.. http://dx.doi.org/10.1890/090047.

[40] Regional CTI Secretariat (2009). Regional plan of action: Coral Triangle initiative on coral reefs, fisheries and food security..

[41] Beger M, Grantham HS, Pressey RL, Wilson KA, Peterson EL (2010). Conservation planning for connectivity across marine, freshwater, and terrestrial realms.. Biological Conservation.

