# Supplementary material for: Prioritizing Land and Sea Conservation Investments to Protect Coral Reefs
Source: PLoS One. 2010 Aug 30;5(8):e12431. doi: 10.1371/journal.pone.0012431 (PMC2930002; doi:10.1371/journal.pone.0012431)
Supplement: Table S1 — Cost and protected area data for coastal catchments and coral reefs in each ecoregion. (0.04 MB DOC) [file pone.0012431.s001.doc]

**Table S1. Cost and protected area data for coastal catchments and coral reefs in each ecoregion.**

| Ecoregion | Annual  management cost  (US $/km^2^) | | Annual  opportunity cost  (US $/km^2^) | | Available  (km^2^) | |
| --- | --- | --- | --- | --- | --- | --- |
|  | Land | Sea | Land | Sea | Land | Sea |
| A, Celebes Sea | 5,618 | 40,320 | 9,075 | 7,548 | 17,176 | 332 |
| B, Solomon Islands | 1,887 | 18,447 | 3,434 | 32,264 | 35,065 | 1,151 |
| C, Bismarck Sea | 2,188 | 15,586 | 7,771 | 8,541 | 184,482 | 1,749 |
| D, Halmahera | 5,618 | 33,236 | 8,206 | 1,775 | 26,628 | 875 |
| E, North Arafura | 5,618 | 33,236 | 23,233 | 1,061 | 6,105 | 908 |
| F, Milne Bay | 1,186 | 15,302 | 7,684 | 19,727 | 71,540 | 3,284 |
| G, SW. Papua | 5,618 | 33,236 | 7,856 | 25 | 22,679 | 1,713 |
| H, Makassar | 5,618 | 33,236 | 8,506 | 2,285 | 140,590 | 2,281 |
| I, Cenderawasih | 5,618 | 33,236 | 7,459 | 248 | 90,451 | 3,356 |
| J, Banda & Molluccas | 5,618 | 33,236 | 10,020 | 2,953 | 106,869 | 3,471 |
| K, Bird's Head | 5,618 | 33,236 | 7,324 | 62 | 69,008 | 4,638 |
| L, N. Lesser Sunda & Savu | 4,751 | 22,660 | 14,387 | 1,829 | 64,642 | 9,771 |
| M, Gulf of Tomini | 5,618 | 33,236 | 9,132 | 144 | 28,681 | 7,925 |
| N, Sulu Sea | 9,293 | 93,262 | 10,279 | 1,813 | 149,194 | 7,400 |
| O, SE. Philippines | 19,110 | 383,483 | 12,321 | 12,979 | 126,004 | 4,647 |
| P, N. Philippines | 19,110 | 383,483 | 11,525 | 1,849 | 82,306 | 12,573 |

*Available land or sea that is not cleared or effectively managed.
