# Supplementary material for: Prioritizing Land and Sea Conservation Investments to Protect Coral Reefs
Source: PLoS One. 2010 Aug 30;5(8):e12431. doi: 10.1371/journal.pone.0012431 (PMC2930002; doi:10.1371/journal.pone.0012431)
Supplement: Table S2 — Ranking results from scenario 1 compared to results from the impact weighting value sensitivity analysis. We perform the analysis with the maximum and minimum impact weighting values provided by experts for the land- and sea-based threats. (0.05 MB DOC) [file pone.0012431.s002.doc]

**Table S2. Ranking results from scenario 1 compared to results from the impact weighting value sensitivity analysis. We perform the analysis with the maximum and minimum impact weighting values provided by experts for the land- and sea-based threats.**

| Ecoregion | ROI,  Scenario 1 | | Max. land and sea | Min. land and sea | Min. land, Max sea | Max. land, Min sea |
| --- | --- | --- | --- | --- | --- | --- |
| A, Celebes Sea, sea | 1 | 1 | | 1 | 1 | 1 |
| B, Solomon Islands, sea | 2 | 2 | | 2 | 2 | 2 |
| C, Bismarck Sea, sea | 3 | 3 | | 3 | 3 | 3 |
| D, Halmahera, sea | 4 | 4 | | 4 | 4 | 4 |
| E, North Arafura, sea | 5 | 5 | | 5 | 5 | 5 |
| F, Milne Bay, sea | 6 | 6 | | 6 | 6 | 6 |
| G, SW. Papua, sea | 7 | 7 | | 7 | 7 | 7 |
| H, Makassar, sea | 8 | 8 | | 8 | 8 | 9 |
| E, North Arafura, land | 9 | 10 | | 9 | 12 | 8 |
| I, Cenderawasih, sea | 10 | 9 | | 10 | 9 | 10 |
| J, Banda & Molluccas, sea | 11 | 11 | | 11 | 10 | 11 |
| K, Bird's Head, sea | 12 | 12 | | 12 | 11 | 13 |
| B, Solomon Islands, land | 13 | 13 | | 13 | 15 | 12 |
| F, Milne Bay, land | 14 | 16 | | 15 | 16 | 14 |
| L, N. Lesser Sunda & Savu, sea | 15 | 14 | | 14 | 13 | 16 |
| A, Celebes Sea, land | 16 | 17 | | 17 | 17 | 15 |
| M, Gulf of Tomini, sea | 17 | 15 | | 16 | 14 | 18 |
| G, SW. Papua, land | 18 | 18 | | 18 | 18 | 17 |
| D, Halmahera, land | 19 | 19 | | 19 | 20 | 19 |
| M, Gulf of Tomin, land | 20 | 20 | | 20 | 21 | 20 |
| N, Sulu Sea, sea | 21 | 21 | | 21 | 19 | 22 |
| L, N Lesser Sunda & Savu, land | 22 | 22 | | 22 | 22 | 21 |
| K, Bird's Head, land | 23 | 23 | | 23 | 24 | 23 |
| C, Bismarck Sea, land | 24 | 24 | | 24 | 25 | 24 |
| I, Cenderawasih , land | 25 | 25 | | 25 | 26 | 25 |
| J, Banda & Molluccas, land | 26 | 27 | | 26 | 27 | 26 |
| O, SE. Philippines, sea | 27 | 26 | | 27 | 23 | 28 |
| H, Makassar, land | 28 | 28 | | 28 | 28 | 27 |
| N, Sulu Sea, land | 29 | 29 | | 29 | 30 | 29 |
| P, N. Philippines, land | 30 | 31 | | 30 | 31 | 30 |
| P, N. Philippines, sea | 31 | 30 | | 31 | 29 | 32 |
| O, SE. Philippines, land | 32 | 32 | | 32 | 32 | 31 |
